# The Story of the Insane from Year to Year

**Published:** 1905-11-25

**Authors:** 


### THE STORY OF THE INSANE FROM YEAR TO YEAR.

Seventeenth Annual Series.

### THREE COUNTIES ASYLUM, NEAR HITCHIN.

On January 1st this Asylum contained 1,017 patients, and on December 31st the number had fallen to 957. There were 149 first admissions, 14 readmissions, 14 transfers, 67 recoveries, 44 discharged not improved, and 117 deaths. The average number resident during the year was 987, and the total number under treatment was 1,194. Calculated on the admissions, the recoveries stand at 37.8 per cent., and calculated on the average number resident the deaths stand at 11.8 per cent. Of the year's deaths 26 were caused by cerebral and spinal diseases, 20 by consumption, 13 by pneumonia, 4 by other lung diseases, and 4 by colitis. Of the year's admissions the insanity was caused by various moral causes in 51 cases, and by physical causes in 190. Among the latter hereditary influence comes first with 48 instances, previous attacks by 30, and intemperance in drink accounts for 18. It will be seen from the death tables that the deaths from lung diseases were 37, of which number 20 were from phthisis. This must, of course, represent a much higher number under treatment for consumption, and we should like to have had some statement from Dr. de Lisle on the matter. Is there any attempt made to separate the sick from the healthy, either by residence in a detached block or in separate wards? Regarding the colitis, Dr. de Lisle is at a loss how to explain the invasion of the disease. There has been no overcrowding, and the sanitary condition of the Asylum is good; but he mentions, "perhaps as a coincidence, that it has apparently made itself known since the introduction of staining the floors and dry rubbing." Altogether there were 15 cases of this troublesome disease, and, excepting two, every ward in the Asylum was attacked. A pension of £26 was granted to Carpenter Sharman, and one of £20 16s. to Gasman G. Roberts. The Committee "thank Dr. de Lisle for the excellent service he has rendered to the Asylum." The cost per head per week was 9s. 10d.

### STIRLING DISTRICT ASYLUM.

In this asylum the numbers were the same at the end of the year as at the beginning, namely, 684. There were 202 first admissions, 53 readmissions, 89 recoveries, 72 discharged relieved, and 92 deaths. The average number resident was 680, and the total number under treatment was 926. The recoveries of the year were 34.9 per cent. of the admissions, and the deaths were 9.7 per cent. of the average number resident. Of the deaths 32 were caused by cerebral and spinal diseases, 19 by consumption, 5 by pneumonia, and 1 by enteritis. Mental worry appears as the cause of the insanity in 4 of the admissions, intemperance in drink in 39, hereditary tendency in 42, and previous attacks in 29. Dr. Robertson's report contains much that is both instructive and interesting. When speaking of alcoholism as a cause of insanity he says that there is no single factor which sends more men insane than over-indulgence in alcohol, and "as it is a cause over the action of which there can be exercised an absolute control, it appears little short of criminal to those in whose minds a health conscience has developed, to allow this evil to exist unchecked by the State." It is pointed out that women suffer less from alcoholic insanity than men, although they are more susceptible of the injurious effects of alcohol, and Dr. Robertson suggests that the comparative freedom may be due either to different customs, fewer opportunities, the possession of less money, or the exercise of more self-control. With men it is too often the custom when friends meet to "have a drink." With women this is unknown, and the sooner men give up this miserable habit the better it will be. As a matter of fact the custom is dying, and will soon be confined to the lower orders. Regarding consumption in asylums, we are informed that it is "the clear duty of those who are responsible for the welfare of the insane in asylums where consumption is prevalent to take those steps which are called for to check the spread of this infectious but preventible disease." This is one of the asylums in which nurses are employed in several of the male wards. For instance, in the sick-room we notice that there are 33 patients, and that these are in charge of one sister, one charge nurse, and three under nurses. In the chronic infirm wards there are 95 patients, one charge nurse, and eight under nurses. Altogether the male patients number 377, the attendants 21, the nurses in the male wards 15. Dr. Robertson tells us that "quite an appreciable change is coming over the behaviour of the nurses, who are rising to a higher conception of their position and duties." On looking over the staff list we find there are three assistant medical officers, two of whom are ladies (one lady doctor in every asylum we have always held to be highly desirable). There is a matron, five assistant matrons, and a lady night superintendent. There were five clinical clerks, of whom two were ladies. There were 85 nurses and only 48 attendants. The old familiar "head attendant" has no place in the list. The house secretary is a lady, but the house steward is a male, and he must feel like Campbell's Last Man awaiting his turn among the skeletons of nations. The committee say that the institution "continues to be maintained in a most efficient manner, thanks to the excellent management of Dr. Robertson." The annual rate of maintenance is £27 10s. per head.

